# Integrating Transcriptomics, Network Pharmacology, and Machine Learning to Reveal Transglutaminase 2 (TGM2) as a Key Target Mediating Taurocholate Efficacy in Colitis

**DOI:** 10.3390/genes16091024

**Published:** 2025-08-29

**Authors:** Junhong Zhu, Huijin Jia, Lanlan Yi, Guangyao Song, Pengfei Fu, Wenjie Cheng, Yuxiao Xie, Wenzhe Shi, Sumei Zhao

**Affiliations:** 1 Yunnan Provincial Key Laboratory of Animal Nutrition and Feed, Faculty of Animal Science and Technology, Yunnan Agricultural University, Kunming 650201, China; junhong-zhu@foxmail.com (J.Z.); 2022210413@stu.ynau.edu.cn (H.J.); yilanlan0217@163.com (L.Y.); songguangyao1990@163.com (G.S.); fupengfeinz151@163.com (P.F.); cwj210365@163.com (W.C.); xieyuxiao@zync.edu.cn (Y.X.); 18287473985@163.com (W.S.); 2 College of Biology and Agriculture, Zunyi Normal University, Zunyi 563006, China

**Keywords:** ulcerative colitis, taurocholate, network pharmacology, machine learning, molecular docking

## Abstract

Background: Ulcerative colitis (UC) is a chronic inflammatory disease of the colon with a rising global incidence. Natural conjugated taurocholic acid (TCA) possesses anti-inflammatory properties and shows potential therapeutic effects against UC, although the underlying mechanisms remain unclear. Methods: This study employed an integrative approach—combining network pharmacology, bioinformatics, machine learning, immune infiltration analysis, and molecular docking—to investigate the therapeutic mechanisms of TCA in UC. UC-related gene expression datasets were obtained from the Gene Expression Omnibus (GEO) database, and potential TCA targets were predicted using the Comparative Toxicogenomics Database (CTD) and TargetNet platforms. Differentially expressed genes (DEGs) were identified and analyzed via GO and KEGG enrichment analyses. Results: Four machine learning algorithms (XGBoost, RF, SVM, and NNet) were used to identify six hub genes (*TGM2*, *MMP9*, *ABCB1*, *NOS2*, *ABCG2*, *CASP1*), which were further validated using an artificial neural network. Immune infiltration analysis with CIBERSORT revealed significant alterations in immune cell populations in UC tissues. Further validation through an artificial neural network model confirmed their predictive ability. The enrichment analysis of the hub genes highlighted their roles in immune-related pathways, while the immune infiltration analysis indicated significant differences in immune cell populations between ulcerative colitis tissues and control tissues. The molecular docking results showed that the binding energies of these six proteins to TCA were lower than −5 kcal/mol, with TGM2 having the strongest binding affinity (−10 kcal/mol). The intervention of TCA on colitis mice could improve the inflammatory response by regulating the expression of the *TGM2* gene. Conclusions: In conclusion, this study suggests that taurocholate alleviates ulcerative colitis by targeting key genes such as *TGM2* and modulating immune-related pathways, providing a novel basis for future therapeutic exploration.

## 1. Introduction

In 2021, inflammatory bowel diseases (IBD) were responsible for 375,140 new cases and a total of 3.83 million cases globally, highlighting the widespread and serious nature of IBD [1]. Ulcerative colitis (UC), a chronic inflammatory disease of the colon and rectum with an unknown etiology, has emerged as a significant global health burden, with high incidence rates in developed countries and a marked increase in incidence in developing countries [2]. Biologic agents and small oral molecules are currently the cornerstone of UC management [3]. However, the limitations of these therapeutic strategies, including adverse side effects, modest improvements in survival rates, vulnerability to drug resistance, and the significant economic burden, suggest that the current clinical management of UC remains suboptimal [4]. Consequently, there is a pressing need for the discovery and development of novel therapeutic candidates for UC that offer reliable efficacy, minimal toxicity, and favorable cost-effectiveness. Recent literature suggests that bile acids possess a range of beneficial properties, including anti-inflammatory, anti-aging, cancer treatment-enhancing, and anti-obesity effects [5,6,7,8].

The biosynthesis of bile acids begins in the liver, where cholesterol acts as the primary precursor and undergoes a series of enzymatic transformations. Bile acids are predominantly found as bile salts, which play a vital role in the digestion and absorption of dietary fats. Primary bile acids, such as cholic acid, are synthesized in hepatocytes and subsequently stored in the gallbladder. Bile acids participate in enterohepatic circulation, where the majority are reabsorbed from the portal vein and returned to the liver, thereby restricting their entry into systemic circulation. Studies have demonstrated that certain bile acids, including taurine-conjugated deoxycholic acid (tauroursodeoxycholic acid) and ursodeoxycholic acid, exhibit anti-inflammatory effects in models of TNBS-induced UC [9,10,11]. Research by Yang et al. found that the Tauroursodeoxycholate significantly alleviated weight loss in mice with ulcerative colitis and improved both colon length and weight [12]. Our research group has also observed that taurocholate (TCA) regulates intestinal barrier function in Dahe pigs and Dahe black pigs. These findings suggest that TCA may offer potential therapeutic benefits for human colitis, although the key targets and underlying molecular mechanisms remain to be fully elucidated. Further investigations are needed to explore these aspects in greater detail.

Machine learning, particularly through advanced models such as ensemble learning and deep learning, is becoming increasingly prevalent in scientific research [13,14]. These algorithms play a crucial role in enhancing performance when analyzing nonlinear and multidimensional data. Supervised machine learning algorithms, including K-nearest neighbor (KNN), support vector machine (SVM), random forest (RF), Extreme Gradient Boosting (XGBoost), and neural networks (NNET), have demonstrated their effectiveness in analyzing complex genomic data [15]. Molecular docking, a computational technique, simulates the interaction between ligands (small molecules) and receptors (biological macromolecules), allowing for the prediction of binding modes, interaction distances, and forces, thereby improving experimental efficiency [16].

In this study, we employed a network pharmacology-based approach to identify the pharmacological interaction network between TCA and UC. Additionally, several machine learning techniques were utilized to identify the hub genes associated with the anti-UC effects of TCA. To further validate our findings, molecular docking analysis and animal experiments were conducted. Our results provide compelling evidence for the potential anti-UC mechanism of TCA at both the molecular and pathway levels, offering novel insights into its potential application for UC treatment.

## 2. Materials and Methods

### 2.1. Acquisition of Relevant Targets of TCA

The canonical SMILES representations of TCA were retrieved from the PubChem database (https://pubchem.ncbi.nlm.nih.gov/, accessed on 16 November 2024). These SMILES strings were subsequently utilized for target protein prediction using the Comparative Toxicogenomics Database (https://ctdbase.org//, accessed on 16 November 2024) and TargetNet (http://targetnet.scbdd.com/, accessed on 16 November 2024) [17,18]. The resulting gene identifiers were mapped to corresponding UniProt IDs using the UniProt ID mapping service (https://www.uniprot.org/, accessed on 16 November 2024) [19]. Finally, targets obtained from both prediction platforms were consolidated, and duplicates were removed to yield the final set of TCA targets.

### 2.2. Acquisition of UC-Related Targets

We searched the UC dataset from the Gene Expression Omnibus (GEO) database (http://www.ncbi.nlm.nih.gov/geo/, accessed on 16 November 2024) using the keyword “Ulcerative colitis”. The datasets utilized in this bioinformatic analysis were all derived from human patient samples. As the training dataset obtained from our retrieval, the GSE75214 dataset contained data from 74 UC samples and 11 healthy control samples [20]. Two independent validation datasets were obtained: GSE73661, containing 67 UC samples and 12 healthy controls [21], and GSE87466, containing 87 UC samples and 21 healthy controls [22]. All the data analyzed in this study were extracted from the GEO database; thus, no ethical approval or informed consent was required. In the next step, we used R 4.4.1 to normalize the above data and then identified the differentially expressed genes (DEGs) associated with UC using two criteria: |log2 fold change (FC)| ≥ 1 and *p*-value < 0.05.

### 2.3. Potential Target Prediction of TCA in UC Treatment

By using a Venn diagram drawn with R 4.4.1, we intersected those previously described DEGs, and the targets of TCA to obtain several intersecting target genes that were predicted to be potential targets of TCA in UC treatment.

### 2.4. Functional Enrichment Analysis

To explore the possible biological functions and main signaling pathways in UC treatment with TCA, we conducted Gene Ontology (GO) and Kyoto Encyclopedia of Genes and Genomes (KEGG) pathway enrichment analyses on the intersecting targets using the ClusterProfiler package in R software 4.4.1. After filtering with the criterion of q-value < 0.05, we ranked the qualifying terms in descending order according to their enrichment scores.

### 2.5. Determination of Hub Genes with Machine Learning

Four machine learning algorithms, XGBoost, RF, SVM, and NNet, were used to further identify the hub genes among the intersecting target genes. To ensure model generalizability, we applied a repeated 10-fold cross-validation strategy during training. In addition, two independent validation datasets (GSE73661 and GSE87466) were used to further evaluate model performance across different cohorts. The residuals of XGBoost, RF, SVM, and NNet models were analyzed using the R software package “DALEX,” and their classification performance was assessed with the Area Under the Curve (AUC) score calculated by the “pROC” package (version 1.18.5). The sub-model with the lowest residual error and the highest AUC score was identified as the optimal prediction model. The genes included in this optimal model were recorded as characteristic genes for further analysis. Finally, we determined the hub genes by computing the intersection of the genes identified with the machine learning algorithms in the TCA–UC interaction, which was followed by gene correlation analysis and differential expression analysis.

### 2.6. *Construction of Artificial Neural Network Model*

An artificial neural network (ANN) is a mathematical model or calculation model that imitates the structure and function of a biological neural network. To evaluate the overall prediction performance of key genes, we established an ANN model using the R package neuralnet (version 1.44.2). The ANN model, which includes an input layer, one or more hidden layers, and an output layer, was constructed based on the selected key genes in the training set.

### 2.7. Gene Set Enrichment Analysis (GSEA)

In order to further clarify the biological function and signal pathway of key genes, based on MSigDB database (https://www.gsea-msigdb.org/gsea/msigdb, accessed on 16 November 2024), Select “c2.cp.v2023.2.Hs.symbols.gmt” and “c5.all.v2023.2.Hs.symbols.gmt” as reference gene sets, and use “corrplot”(v 0.92) package to calculate the Spearman correlation coefficient of each key gene and all other genes, respectively, and then calculate all the genes according to the correlation coefficient. Then, the r package “ClusterProfiler” (v 4.8.3) was used for GSEA (*p* adjust < 0.01).

### 2.8. Methods for Immune Infiltration Analysis

We utilized the CIBERSORT algorithm (https://cibersortx.stanford.edu/, accessed on 16 November 2024) for immune infiltration analysis, with the aim of evaluating the potential link between the target hub genes and changes in the immune microenvironment of patients with UC. After estimating the relative proportion of 22 types of immune cells in each sample from the GSE75214 dataset, the immunological scores of these samples were calculated using the ESTIMATE algorithm. In addition, the correlation between the hub genes and the immune cells was determined by performing Spearman correlation analysis.

### 2.9. Chromosome Localization

Studying the distribution of related genes on chromosomes can provide insights into genome structure and function, reveal the relationship between genetic variation and disease occurrence, and offer new directions for disease diagnosis and clinical treatment. To determine the chromosomal positions of candidate key genes, we utilized the Circos software (v 1.2.2) tool to visualize their distribution across human chromosomes. This visualization allows for a clear representation of gene locations and their interrelationships, providing a foundation for further functional analysis and investigation of disease mechanisms.

### 2.10. Molecular Docking Verification

To evaluate the binding affinity of the compound TCA with the key genes, we conducted molecular docking studies using CB-Dock 2 (https://cadd.labshare.cn/cb-dock2/index.php, accessed on 16 November 2024) and visualized the results with PyMOL (Version 3.1). Initially, we retrieved the three-dimensional structures of key genes by performing an advanced structure search (Accessed on 16 November 2024, https://www.rcsb.org/search/advanced/structure), and the corresponding PDB IDs for the proteins are as follows: TGM2 (PDB ID: 3LY6), MMP9 (PDB ID: 1L6J), ABCB1 (PDB ID: 6FN4), NOS2 (PDB ID: 2NSI), ABCG2 (PDB ID: 5NJ3), CASP1 (PDB ID: 1IBC). Next, we obtained the molecular structure of TCA (CID: 6421) from the PubChem database (Accessed on 16 November 2024, https://pubchem.ncbi.nlm.nih.gov/). The TCA molecule and the three-dimensional structures of key genes were then imported into CB-Dock 2, and molecular docking was performed against each target protein using the default docking protocol and parameters. After the docking process, the best docking model is selected according to the model value with the largest binding energy, and the interaction is analyzed in detail. Visualization and rendering of the final images were carried out using PyMOL and LigPlus.

### 2.11. Validation of Experimental Protocols in Mice

Male 5-week-old C57BL/6 mice were purchased from Yunnan Zhili Technology Co. (Qujing, China) and housed in a temperature-controlled room (22 ± 2 °C, 12 h light/dark cycle) with free access to food and water. Five-week-old mice were chosen because they are young adults with stable growth and mature immune and gastrointestinal systems, minimizing inter-individual variability. Male mice were used to avoid potential effects of the estrous cycle on immune and intestinal responses, ensuring experimental consistency. All animal breeding, maintenance, and experimental procedures were conducted in accordance with the guidelines established by the Institutional Use Committee of Yunnan Agricultural University under a Project License (SYXK (Dian) K2020-0005).

Thirty-two mice were randomly divided into four groups (n = 8 per group) and fed a maintenance diet. The CON and LPS groups were gavaged daily with 0.1 mL normal saline, while the T40 and TL40 groups were gavaged with taurocholate (40 mg/kg body weight) daily. The pre-test period lasted for 7 days, followed by a 14-day main experimental period. After fasting for 12 h, the CON and T40 groups were intraperitoneally injected with 0.5 mL sterile saline, whereas the LPS and TL40 groups received lipopolysaccharide (LLPS, Sigma-Aldrich, 2 Science Park Drive, Ascent Building, Singapore) at 10 mg/kg body weight diluted in 0.5 mL saline. Three hours after injection, mice developed acute intestinal inflammation, consistent with previously reported protocols for colitis induction.

For the evaluation of ulcerative colitis (UC), we followed standard UC scoring methods, including assessments of body weight loss, stool consistency, intestinal morphology, and colon damage [23]. After euthanasia, colon tissues were collected for RNA extraction and gene expression analysis. Total RNA was extracted from colon tissues using TRIzol reagent (Invitrogen, Carlsbad, CA, USA) according to the manufacturer’s instructions. RNA purity and concentration were measured with a NanoDrop 2000 spectrophotometer (Thermo Fisher Scientific, Waltham, MA, USA), and 1 μg RNA was reverse-transcribed into cDNA using the PrimeScript RT reagent kit (Takara, Tokyo, Japan). Quantitative real-time PCR (qRT-PCR) was performed using TB Green Premix Ex Taq II (Takara, Japan) on a CFX96 Real-Time PCR Detection System (Bio-Rad, Hercules, CA, USA). Each reaction was conducted in triplicate in a total volume of 20 μL. GAPDH was used as the internal control, and relative expression levels were calculated using the 2^−ΔΔCt^ method. The primer sequences are provided in Table S1.

### 2.12. Statistical Analysis

Statistical analysis was performed using R(v4.0.2) and GraphPad Prism(v8.4.0). The Wilcoxon rank-sum test. All statistical tests were two-sided, and significant differences between each two groups were indicated by * *p* < 0.05 and ** *p* < 0.01.

## 3. Results

### 3.1. Acquisition of TCA Targets and Disease Targets and Functional Enrichment Analysis of Intersecting Genes

The standardized SMILES (Simplified Molecular Input Line Entry System) structures of TCA (trans-4-hydroxycinnamic acid) were initially retrieved from the PubChem database (https://pubchem.ncbi.nlm.nih.gov/, accessed on 16 November 2024). Afterward, we imported these SMILES files into the Comparative Toxicogenomics Database (CTD) (https://ctdbase.org//, accessed on 16 November 2024) and the TargetNet (http://targetnet.scbdd.com/, accessed on 16 November 2024) to predict the corresponding target proteins for TCA. These genes’ Uniprot IDs were then interpreted using the ID mapping tool of the Uniprot database (https://www.uniprot.org/, accessed on 16 November 2024). At last, we combined the targets’ information mentioned above and obtained the targets of TCA after removing any duplicates.

**Figure 1:**
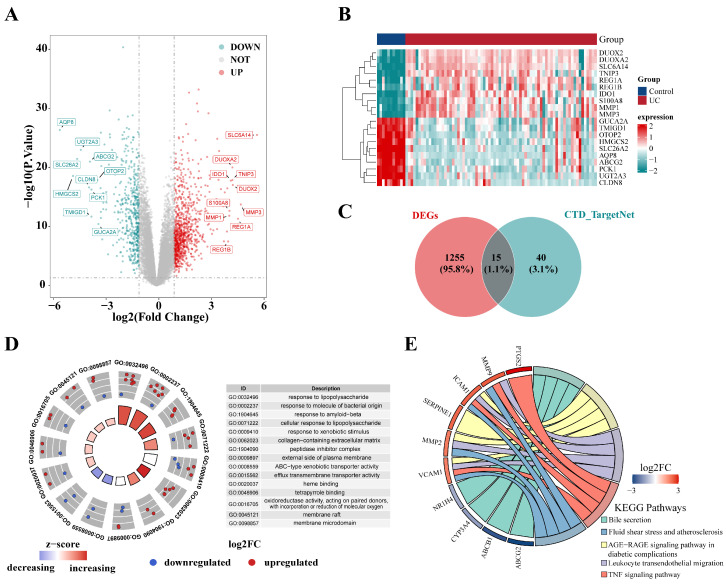
Identification of hub targets and functional enrichment analysis. (**A**) Volcano plot of the DEGs; (**B**) Heat map of the 20 most significant DEGs; (**C**) Venn diagram of hub target identification; (**D**) GO Enrichment analysis of hub genes represented in a paired radar chart; (**E**) KEGG pathway enrichment analysis of hub genes visualized in a chord diagram.

By searching the CTD and the TargetNet Target Prediction database, we found that TCA was associated with 888 target genes (Table S2). Further analysis shows that there are 55 intersecting genes in the two databases. Before performing differential expression analysis, we normalized the UC dataset to eliminate the batch effect. By performing differential expression analysis on the GSE75214 dataset, we discovered 1203 DEGs. The data were visually represented using a volcano plot and heat map (Figure 1A,B; Table S1). Fifteen key genes were identified by computing the intersection of DEGs and the target genes of TCA (Figure 1C; Table S1). GO and KEGG pathway enrichment analyses were performed on these 15 intersecting target genes. The results of the GO analysis are presented in Figure 1D. The potential target genes were mainly enriched in the following biological process (BP) terms: response to lipopolysaccharide (GO:0032496), response to molecule of bacterial origin (GO:0002237), response to amyloid-beta (GO:1904645), cellular response to lipopolysaccharide (GO:0071222), response to xenobiotic stimulus (GO:0009410). The enriched cellular component (CC) entries included the collagen-containing extracellular matrix (GO:0062023), peptidase inhibitor complex (GO:1904090), external side of plasma membrane (GO:0009897), membrane raft (GO:0045121), and membrane microdomain (GO:0098857). The enriched molecular function (MF) terms included ABC-type xenobiotic transporter activity (GO:0008559), efflux transmembrane transporter activity (GO:0015562), heme binding tetrapyrrole binding oxidoreductase activity (GO:0020037), acting on paired donors (GO:0046906), with incorporation of or reduction in molecular oxygen (GO:0016705). In addition, according to the KEGG pathway enrichment analysis, these intersecting target genes were primarily enriched in signaling pathways such as the Bile secretion, Fluid shear stress and atherosclerosis, AGE-RAGE signaling pathway in diabetic complications, Leukocyte transendothelial migration, and TNF signaling pathway (Figure 1E).

### 3.2. Determination of Target Hub Genes with Machine Learning

**Figure 2:**
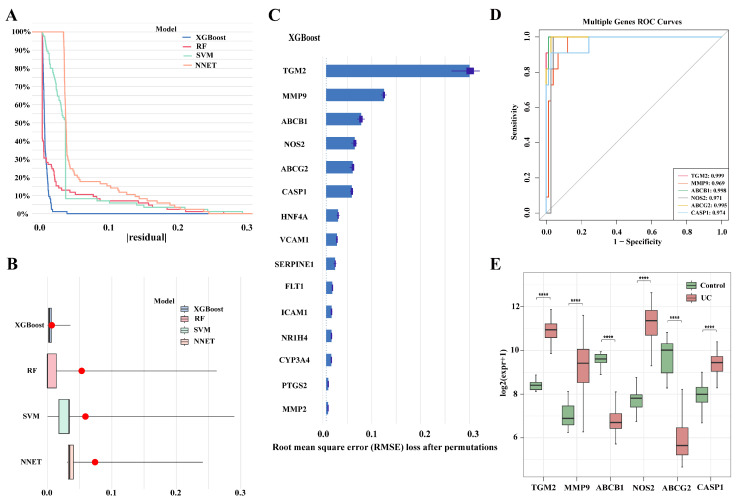
Evaluation of model performance and characteristic gene expression in the training set. (**A**) Cumulative residual distribution of the samples; (**B**) Boxplot of sample residuals, with the red dots representing the root mean square of residuals; (**C**) Feature importance of explanatory variables in the XGBoost model; (**D**) ROC curve analysis of characteristic genes in the training set; (**E**) Evaluation of the expression levels of characteristic genes in the training set. Note: **** *p* < 0.0001.

In order to further screen candidate genes, based on the 15 candidate genes obtained above, we first established RF, XGBoost, SVM, and NNet models in the training set using the R package “CARET” (v6.0-94). The residuals of the four models were analyzed using the R package “DALEX” (v2.4.3), and we found that XGBoost exhibited the smallest residuals and the best model fitting (Figure 2A,B). To ensure model robustness, we applied a 10-fold cross-validation strategy, and the models showed consistent performance with stable AUC and accuracy values. The detailed performance metrics, including Accuracy, Sensitivity, Specificity, and AUC for all models on both the training and independent validation datasets, are summarized in Table S3. Furthermore, independent validation using the dataset (GSE73661 and GSE87466) confirmed the predictive ability of the models, supporting their robustness and reducing the likelihood of overfitting. According to the importance ranking of genes in the XGBoost model, six target hub genes were identified: Transglutaminase 2 (TGM2), Matrix metalloproteinase 9 (MMP9), ATP binding cassette subfamily B member 1 (ABCB1), Nitric oxide synthase 2 (NOS2), ATP binding cassette subfamily G member 2 (ABCG2), and Caspase 1 (CASP1) (Figure 2C). Receiver Operating Characteristic (ROC) curve analysis of these six key genes showed that the AUC values of all characteristic genes were greater than 0.95 (Figure 2D). Moreover, the expression levels of ACBC1 and ABCG2 in control tissues were significantly higher than those in UC tissues, whereas the expression levels of TGM2, MMP9, NOS2, and CASP1 in control tissues were significantly lower than those in UC tissues (Figure 2E).

### 3.3. Evaluation of Artificial Neural Network Performance

**Figure 3:**
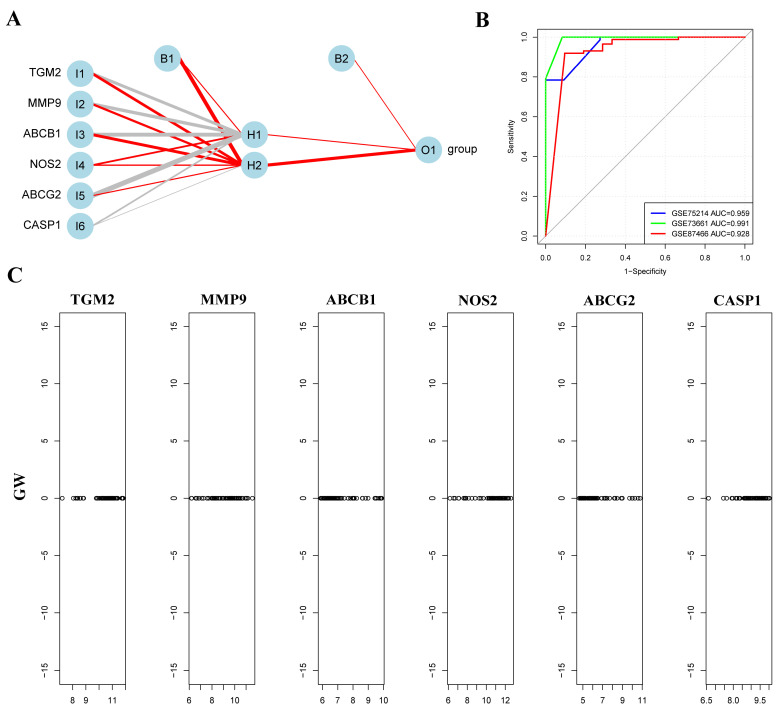
Architecture and performance evaluation of the ANN model for gene classification. (**A**) Structure of the ANN model, including input layers (I1 to I6), biases (B1 and B2), hidden layer nodes (H1 and H2), and output nodes (O1). Gray lines represent negative weights, red lines represent positive weights, and line thickness corresponds to the magnitude of the coefficients. (**B**) ROC curve for the neural network model, with the blue curve representing the model’s performance, achieving an AUC of 1, indicating perfect classification ability. (**C**) Contribution-weight plots for each gene in the neural network model. The x-axis shows the expression levels of individual genes, while the y-axis represents their respective contribution weights (GW). Each subplot corresponds to one gene (*TGM2*, *MMP9*, *ABCB1*, *NOS2*, *ABCG2*, and *CASP1*).

ANN is a mathematical model or calculation model that imitates the structure and function of a biological neural network [24]. A neural network is calculated by a large number of artificial neurons. In order to evaluate the overall prediction performance of key genes, in the training set, based on the above six key genes, an ANN model is established by using the R package neuralnet (v 1.44.2). The network consists of an input layer with six nodes (corresponding to the six genes), one hidden layer with two neurons, and an output layer for binary classification. The neural network was trained using a 5-fold cross-validation strategy to ensure robust performance estimation. The mean validation accuracy across folds was 0.965, with an AUC of 0.959. Independent validation on the GSE73661 dataset confirmed the robustness of the model, achieving an accuracy of 0.987 and an AUC of 0.991, while validation on the GSE87466 dataset achieved an accuracy of 0.898 and an AUC of 0.928. The results are shown in Figure 3A–C.

### 3.4. Enrichment Analysis of Key Genes

**Figure 4:**
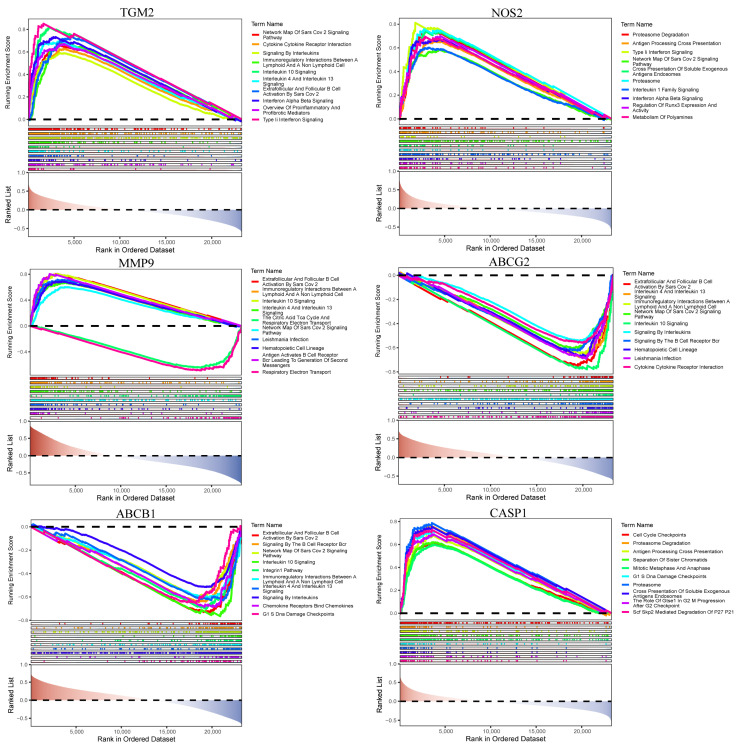
GSEA enrichment analysis of key characteristic genes.

In the single-gene KEGG enrichment analysis, TGM2 was significantly enriched in the SARS-CoV-2 signaling pathway, cytokine receptor interaction, signaling by interleukins, immunoregulatory interactions between lymphoid cells, interleukin 4 and interleukin 13 signaling, extrafollicular and follicular B cell activation by SARS-CoV-2, interferon alpha/beta signaling, an overview of proinflammatory and profibrotic mediators, and Type II interferon signaling. MMP9 was enriched in B cell activation by SARS-CoV-2, immunoregulatory interactions between lymphoid and non-lymphoid cells, interleukin 4 and 13 signaling, the TCA cycle and respiratory electron transport, SARS-CoV-2 signaling pathway, Leishmania infection, hematopoietic cell lineage, and antigen-activated B cell receptor (BCR) signaling. ABCB1 was enriched in B cell activation by SARS-CoV-2, BCR signaling, SARS-CoV-2 signaling pathway, interleukin 10 signaling, the integrin β1 pathway, immunoregulatory interactions, interleukin 4 and 13 signaling, signaling by interleukins, chemokine receptor interactions, and G1/S DNA damage checkpoints. NOS2 was enriched in proteasome degradation, antigen processing and cross-presentation, Type II interferon signaling, SARS-CoV-2 signaling pathway, cross-presentation of soluble exogenous antigen, interleukin 1 family signaling, interferon alpha/beta signaling, regulation of RUNX3 expression, and polyamine metabolism. ABCG2 was enriched in B cell activation by SARS-CoV-2, interleukin 4 and 13 signaling, immunoregulatory interactions, SARS-CoV-2 signaling pathway, interleukin 10 signaling, signaling by interleukins, BCR signaling, hematopoietic cell lineage, Leishmania infection, and cytokine receptor interaction. Finally, CASP1 was enriched in cell cycle checkpoints, proteasome degradation, antigen processing and cross-presentation, sister chromatid separation, mitotic metaphase and anaphase, G1/S DNA damage checkpoints, the role of GTSE1 in G2/M progression, and SCF-SKP2-mediated degradation of p27/p21 (Figure 4).

Although some enriched pathways (e.g., SARS-CoV-2 signaling, IL-4/IL-13 signaling) appear broad, the core modules of these pathways are closely linked to UC pathogenesis. Specifically, IL-4/IL-13 signaling reflects Th2-skewed immune responses, which play a crucial role in UC-related mucosal inflammation and epithelial barrier dysfunction. Likewise, SARS-CoV-2–related pathways capture interferon responses and cytokine-driven inflammation, processes that are also central to UC progression.

### 3.5. Evaluation of Immune Cell Infiltration

**Figure 5:**
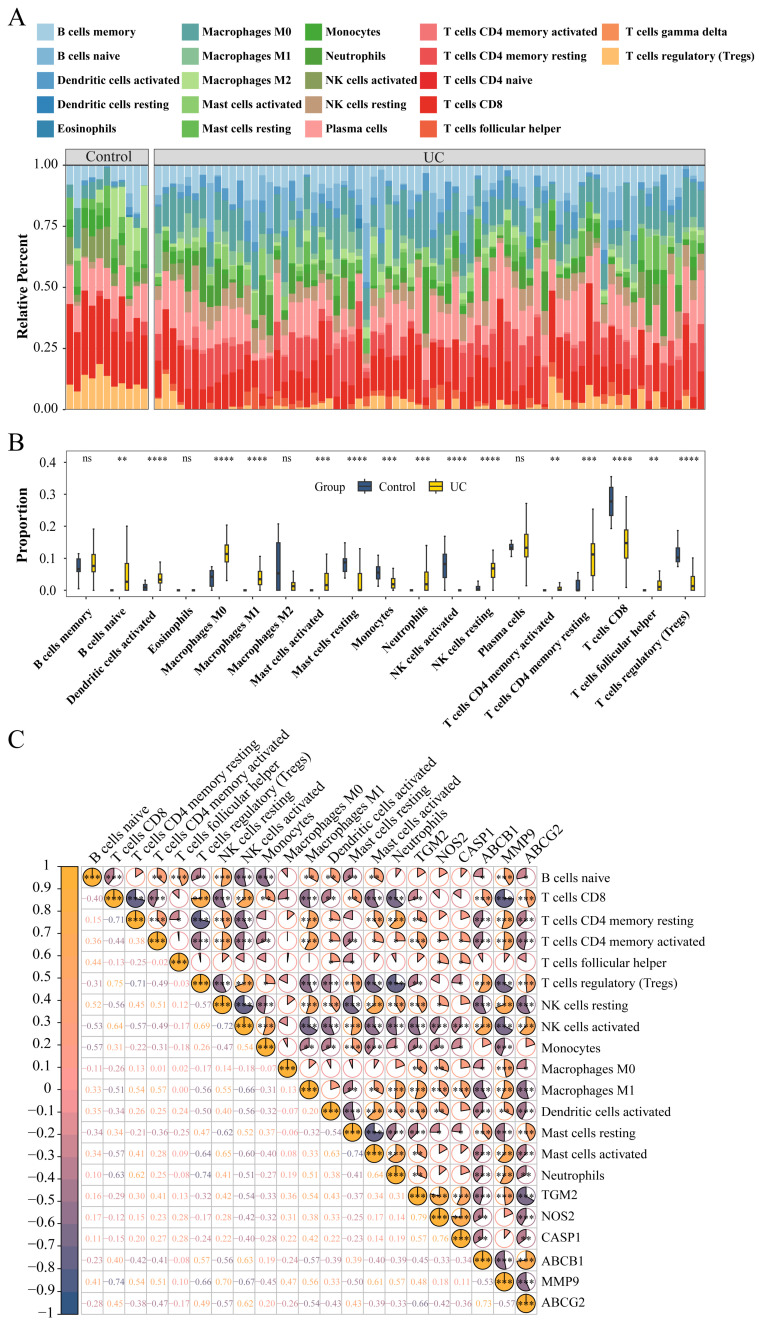
Immune cell infiltration analysis and correlation with hub gene expression in the GSE75214 dataset. (**A**) Stacked column chart showing the infiltration levels of multiple types of immune cells in each sample of the GSE75214 dataset; (**B**) Box plot comparing the infiltration of different immune cells between UC and normal samples; (**C**) Heat map illustrating the correlation between immune cell infiltration and the expression of hub genes. * *p* < 0.05, ** *p* < 0.01, *** *p* < 0.001, **** *p* < 0.0001 and ^ns^ *p* > 0.05.

Firstly, we used cell-type identification by estimating relative subsets of RNA transcripts (CIBERSORT) tool to visualize the infiltration of the 22 immune cells within each sample in the GSE75214 dataset (Figure 5A). The infiltration degree of disease samples is the highest in CD8 T cells. Then, we analyzed the distinctions in the infiltration of the different immune cells in UC tissues and controls (Figure 5B). The results suggested that the proportions of B cells naive, dendritic cells activated, M0 macrophages, M1 macrophages, mast cells activated, NK cells resting, T cells CD4 memory activated, T cells CD4 memory resting, and T cell follicular helper were remarkably higher in UC samples, while those of Mast cells activated, monocytes, NK cells activated, T cells CD8, and T cells regulatory (Tregs) were significantly lower in UC tissues. Furthermore, we analyzed the correlation between the expression levels of the six target hub genes and the infiltration of various types of immune cells (Figure 5C). The outcomes revealed that the relative abundance of B cells naive, T cells CD4 memory resting, T cells CD4 memory activated, NK cells resting, macrophages M0, macrophages M1, dendritic cells activated, mast cells activated, and neutrophils were significantly positively correlated with the expression levels of *TGM2*, *NOS2*, *CASP1*, and *MMP9* genes; on the other hand, the proportion of that was significantly negatively associated with the expression levels of *ABCB1* and *ABCG2* genes. These results indicate that the predicted TCA targets are functionally linked to immune cell recruitment and activation in colitis, thereby supporting their relevance for further structural validation.

### 3.6. Chromosome Localization Analysis

**Figure 6:**
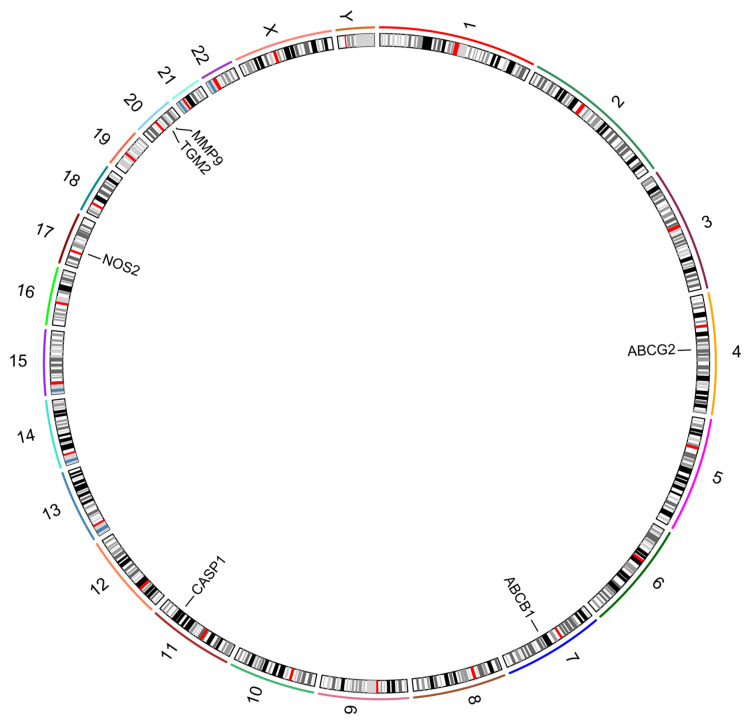
Chromosomal distribution of candidate key genes. Chromosomes are labeled 1–22, X, and Y. Colors represent Giemsa-staining patterns, with dark bands indicating gene-rich regions, light bands indicating gene-poor regions, red bands for centromeres, gray bands for variable regions, and pale bands for stalks.

In order to explore the positional distribution of candidate key genes on the human chromosome, this paper used Circos software for visualization. Circos is a widely used tool for genomic data visualization, which can intuitively present the distribution pattern of genes on the chromosome. Figure 6 demonstrates the distribution of candidate key genes on human chromosomes. The results showed that some candidate genes showed a tendency to cluster in specific regions of the chromosome, especially on chromosome 20, where both the *MMP9* and *TGM2* genes were distributed on this chromosome. In addition, *ABCG2* was distributed on chromosome 4, *ABCB1* on chromosome 7, *CASP1* gene in steps on chromosome 11, and *NOS2* gene on chromosome 17 (Figure 6). Mapping the chromosomal distribution of candidate targets provides genomic context and suggests potential co-regulation, which guided our selection of key targets (*NOS2*, *ABCG2*, *CASP1*) for subsequent molecular docking and expression validation.

### 3.7. Molecular Docking of the Key Compounds and Core Targets

**Figure 7:**
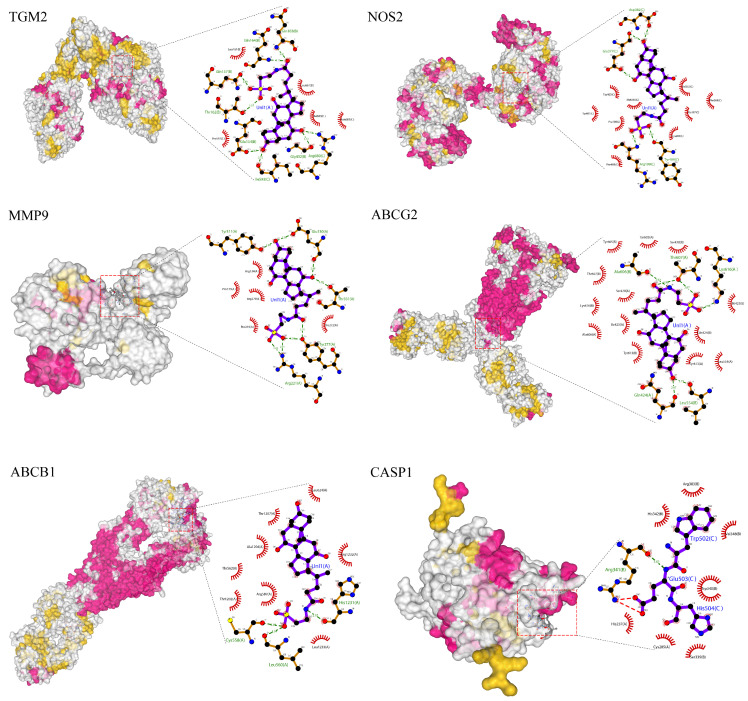
Molecular docking analysis of TCA with the target hub proteins. The predicted binding poses of TCA within the most favorable binding cavity of TGM2, MMP9, ABCB1, NOS2, ABCG2, and CASP1 are shown. Surface colors indicate secondary structural elements: magenta for β-sheets, yellow for α-helices, and white for loop regions. Detailed cavity information and interacting residues are provided in Table S4.

Molecular docking is a computer simulation method of protein-ligand interaction based on induced-fit theory, which is widely used in drug development and mechanism prediction of natural products. In order to further explore the potential action mechanisms of TCA, the binding energies between TCA compounds and core targets were calculated. The docking results showed that the binding energy of TCA-*TGM2* was −10.0 kcal/mol, TCA-*MMP9* was −8.0 kcal/mol, TCA-*ABCB1* was −9.2 kcal/mol, TCA-*NOS2* was −8.7 kcal/mol, TCA-*ABCG2* was −8.9 kcal/mol, and TCA-*CASP1* was −6.5 kcal/mol. All of them were below −5 kcal/mol, indicating that the selected TCA had high binding affinity with the target, among which TCA has the highest binding affinity with *TGM2* (Figure 7).

To further clarify the binding sites of TCA with each target protein, we analyzed the potential binding cavities, including cavity volume, center position, binding energy, and key contact residues (see Table S4). The results showed that the binding of TCA to TGM2 is located near its catalytic domain (Cavity 1, volume 14493 Å^3^, binding energy −10.0 kcal/mol), which may affect its transamidase activity; the binding to MMP9 is situated in its zinc-binding region (Cavity 1, volume 1187 Å^3^, binding energy −8.0 kcal/mol), potentially interfering with its protease function; the binding to ABCB1 and ABCG2 occurs within their transmembrane transport regions (Cavity 1 for both, binding energies −9.2 and −8.9 kcal/mol, respectively), which could influence their efflux pump functions; the binding to NOS2 is located in its oxygenase domain (Cavity 1, volume 9867 Å^3^, binding energy −8.7 kcal/mol), potentially regulating NO synthesis; whereas the binding to CASP1 is at the interface of its p20/p10 subunits (Cavity 1, volume 421 Å^3^, binding energy −6.5 kcal/mol). Although the binding energy is relatively lower, its biological significance requires further validation.

### 3.8. Validation Experiment of the Key Compounds and Core Targets

**Figure 8:**
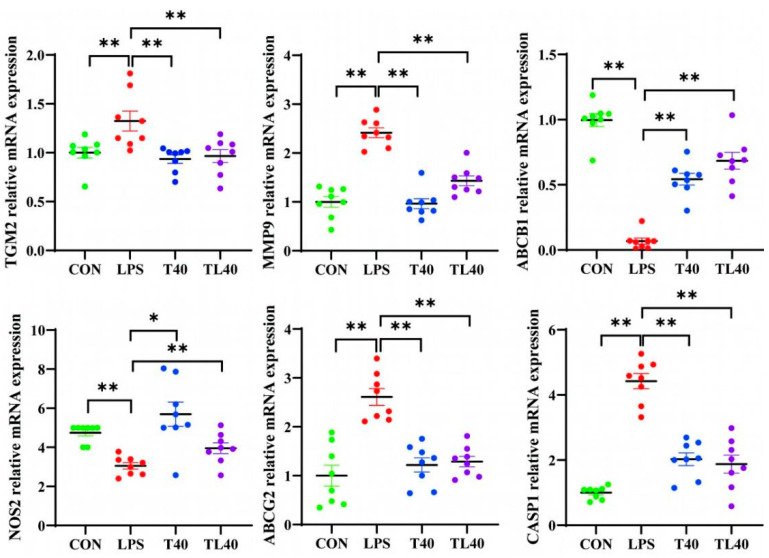
Effects of TCA on gene expression levels in mouse colon tissues. Data are expressed as mean ± SD (n = 8). CON: control group; LPS: lipopolysaccharide-induced inflammation group; T40: taurocholate (40 mg/kg) gavage group; TL40: taurocholate (40 mg/kg) + lipopolysaccharide group. * *p* < 0.05, ** *p* < 0.01 vs. CON group. Different colors represent different groups.

In vivo experiments were performed to validate the potential molecular mechanisms of TCA predicted by the network pharmacology analysis. The mRNA expression levels of *TGM2*, *MMP9*, *ABCG2*, and *CASP1* genes in the LPS group were significantly higher than those in the other three groups (*p* < 0.01). There was no significant difference between the T40 and TL40 groups and the CON group (*p* > 0.05). The mRNA expression levels of *ABCB1* and *NOS2* genes in the LPS group were significantly lower than those in the other three groups (*p* < 0.01). There was also no significant difference between the T40 and TL40 groups and the CON group (*p* > 0.05) (Figure 8).

## 4. Discussion

The incidence of colitis, as a common digestive system disease, is increasing year by year, and it has become an important disease that seriously affects people’s quality of life and physical and mental health [25]. Especially UC, its symptoms are mainly diarrhea, bloody stool, and abdominal pain, often accompanied by systemic symptoms such as weight loss and fatigue. The symptoms of the disease are more serious in the acute attack period, but they are relieved in the remission period. The course of the disease is easy to repeat, which poses a threat to the life and health of patients and has attracted widespread attention. Although related literature has reported that intestinal microorganisms, immune response, and oxidative stress may be potential therapeutic targets for colitis, drug therapy is still the main means to treat colitis [26,27]. Glucocorticoids and 5-aminosalicylic acid drugs are commonly used to treat colitis [28,29]. They can relieve symptoms by regulating the immune response and inhibiting intestinal inflammation. However, many patients will develop drug resistance after long-term medication and even aggravate their condition. Our research group found that TCA also has a certain effect in treating the colon. As a natural conjugated bile acid, it has no drug resistance, and the study on its pathological mechanism has become a hot and difficult point in the field of gastroenterology.

In this study, with the help of CTD, TargetNet, and GEO database analysis, it was found that 15 signaling pathways in diabetic complications, Leukocyte transends were involved in the process of TCA treating UC, and were preliminarily enriched in signaling pathways such as the bile secret. Fluid shear stress and atherosclerosis, AGE-RAGE signaothelial migration, and TNF signaling pathway. The enrichment of these pathways reveals that TCA may play a role in the treatment of UC through various mechanisms. Firstly, the signal pathway related to bile secretion and atherosclerosis may reflect that TCA improves the inflammatory state of ulcerative colitis by regulating the intestinal microenvironment and metabolic pathway [30,31]. Secondly, the relationship between the AGE-RAGE signaling pathway and diabetic complications suggests that TCA may alleviate the inflammatory response of UC by alleviating oxidative stress and glycosylation-related injuries [32]. In addition, the involvement of leukocyte migration across the endothelium and the TNF signaling pathway further shows that TCA may reduce the migration of immune cells and the release of inflammatory factors by regulating immune response, thus alleviating the symptoms of UC [33]. In the present research, machine learning is more and more widely used in the screening of key genes, metabolites, and microorganisms. Building upon the identified signaling pathways through which TCA may exert its effects, we further employed machine learning methods to screen for key genes, aiming to elucidate their potential mechanisms of action. Four machine learning algorithms, RF, XGBoost, SVM, and NNet, were employed to identify key genes. RF and XGBoost are ensemble tree-based methods that provide intrinsic measures of feature importance, whereas SVM and NNet can also be applied for gene selection through approaches such as coefficient weight analysis, recursive feature elimination, or interpretation of model weights. The reliability of the model can be evaluated by model residuals and ROC curves [34]. Recently, 101 algorithm combinations of 10 machine learning methods have been studied to screen the model and find the best algorithm mode [35]. In a word, machine learning algorithms are more and more used in scientific research.

In this study, machine learning was employed to further analyze 15 intersecting genes, ultimately identifying six key signature genes. Compared with the control group, the expression levels of *TGM2*, *MMP9*, *NOS2*, and *CASP1* were significantly upregulated in UC samples, while the expression levels of *ABCB1* and *ABCG2* were significantly downregulated. Similarly, in Yang et al.’s study on colorectal cancer, the expression level of *TGM2* was significantly elevated in diseased tissues, which was associated with reduced survival rates in colorectal cancer patients [36]. Miyoshi et al. further demonstrated that *TGM2* serves as a novel prognostic marker and a potential therapeutic target in colorectal cancer. Additionally, it has been reported that *MMP9* plays a critical role in linking CRC with neuropathic conditions, potentially driving neuronal damage in colorectal cancer patients [37]. The upregulation of *NOS2* has been shown to exacerbate acute colitis, aligning with our findings where *NOS2* expression was significantly elevated in ulcerative colitis samples [38]. Moreover, *CASP1* has been identified as a novel biomarker and a potential target for immunotherapy in colorectal cancer patients [39]. Based on the key genes screened through machine learning, we analyzed their expression levels in UC, reviewed supporting literature, and examined their associations with immune cell infiltration. The results not only reveal the complex immune microenvironment characteristics of UC patients, but also provide us with a new perspective to understand the disease. Among them, CD8 T cells, as one of the important effector T cells, play a key role in the fight against pathogen infection, and the decrease in CD8 T cells in UC samples may indicate that the body’s ability to fight chronic inflammation is weakened [40,41]. On the other hand, the increase in activated memory CD4 T cells and follicular helper T cells may reflect the body’s attempt to cope with abnormal stimulation in the intestine by enhancing adaptive immune response [42]. At the same time, the increase in activated dendritic cells and macrophages indicates that the innate immune system is also actively involved in the pathological process of UC [43,44]. In addition to differential expression analysis, immune infiltration and chromosomal localization provided complementary insights into the biological relevance of the predicted TCA targets. The immune infiltration analysis highlighted how candidate genes were associated with distinct immune cell populations in colitis, supporting their role in regulating intestinal immune responses. Chromosomal localization further revealed the genomic distribution of these genes, suggesting potential co-regulation and facilitating the prioritization of key immune-related targets. Together, these analyses not only strengthened the immunological and genomic context of the candidate genes but also provided a rationale for selecting specific targets such as *NOS2*, *ABCG2*, and *CASP1* for subsequent docking and expression validation.

To date, no research has been reported on predicting UC and TCA using neural network models. However, in other areas of disease research, numerous studies have employed artificial neural networks to predict cancer risk [45,46,47]. Several studies have collected data from 80 patients with advanced lung cancer requiring chemotherapy and developed multiple prognostic prediction models by screening clinical variables. The results indicate that the artificial neural network model demonstrates high accuracy in predicting pneumonia infections in lung cancer patients undergoing chemotherapy [48]. Similarly, the neural network model for predicting potential CT benefits, developed by Lu J et al., can accurately forecast the potential benefits and long-term prognosis of adjuvant chemotherapy in patients with advanced gastric cancer, demonstrating strong prognostic stratification capabilities [49]. Based on this finding, through self-validation using independent datasets and samples, the neural network model we developed demonstrates strong predictive capability and recognition accuracy for TCA. However, the performance of the ANN model still requires further validation through comparison with other established and reliable computer-based diagnostic models. To further strengthen the reliability of our findings, we implemented a 5-fold cross-validation strategy to minimize overfitting and observed consistent performance across folds. Importantly, independent validation on the GSE73661 dataset confirmed the robustness of the models, supporting their predictive ability and reducing the likelihood of overfitting. Nevertheless, additional external datasets and larger cohorts will be necessary to comprehensively validate the predictive models in future studies. Further molecular docking is consistent with the machine learning results. Molecular docking involves simulating the binding process between drug molecules and target proteins at the atomic level to assess their binding modes and affinities. TCA exhibits a high binding affinity for its target proteins, with the interaction between TCA and TGM2 demonstrating the highest affinity among the observed bindings. The intervention of TCA on colitis mice can improve the inflammatory response by regulating the expression of the *TGM2* gene. Previous studies have identified *TGM2* as a potential therapeutic target in cancer treatment. Moreover, *TGM2* plays a critical role in the repair processes of the liver following prolonged toxic injury. The molecular docking results suggest that the binding sites of TCA for TGM2, MMP9, ABCB1, NOS2, and ABCG2 are all located within their key functional domains, potentially influencing their biological functions through allosteric inhibition or competitive binding. For instance, binding to the catalytic domain of TGM2 might inhibit its cross-linking activity, thereby modulating the inflammatory response; binding to the active site of MMP9 could inhibit its matrix degradation function, alleviating tissue damage. However, although the binding site for CASP1 exhibits certain binding affinity, its location in a non-canonical region and the predominance of structural amino acids among the contact residues suggest that this binding might not specifically regulate CASP1 activation. Further validation through subsequent point mutation or functional experiments is necessary. This targeted interaction with central functional domains provides a plausible structural basis for the therapeutic effects of TCA observed in our animal model and underscores its multi-target mechanism of action. The strong binding affinity for TGM2, in particular, aligns with its identification as the top candidate through machine learning and its significant role in inflammation. While TGM2 does have the lowest docking energy, indicating the strongest binding affinity with TCA, its biological significance extends beyond just its binding strength. TGM2 is a key player in immune response, inflammation, and extracellular matrix repair processes, which are crucial in ulcerative colitis (UC) [50]. It is well-documented in the literature that TGM2 plays an important role in regulating immune responses and tissue repair, particularly in intestinal inflammation and repair [51]. It has been identified as a critical regulator in several pathological processes related to UC and cancer. It is important to note that the observed effects of TCA on both the binding affinity of target proteins and the regulation of their mRNA expression do not represent contradictory mechanisms, but rather complementary modes of action. On one hand, TCA can modulate transcriptional activity through bile acid receptors such as FXR and TGR5, thereby altering the expression of downstream genes, including *TGM2*, *NOS2*, and *ABCG2*. On the other hand, our docking results suggest that TCA may also directly interact with certain proteins, influencing their functional activity post-translationally. Together, these dual mechanisms—gene expression regulation and direct protein binding—provide a more comprehensive framework to explain the multifaceted role of TCA in modulating immune and inflammatory responses in UC.

In comparison, while MMP9 and ABCG2 exhibit higher binding energies in the docking analysis, their roles in UC have not been as extensively explored as TGM2. Additionally, their mechanisms of action are more complex. For example, MMP9 is involved in tissue degradation and remodeling, and its role in local immune responses is more related to the acute phases of UC, without demonstrating the broad immune-modulatory functions seen in TGM2 [52,53]. ABCG2, primarily involved in drug efflux and drug resistance, may influence the biological actions of TCA, but its direct involvement in UC treatment mechanisms is less pronounced [54]. Therefore, we believe that TGM2’s low docking energy is not only linked to its strong binding affinity but also to its significant biological function in UC and other immune-mediated diseases. Based on our findings, *TGM2* is involved in the regulation of UC by TCA, highlighting its multifaceted role in disease modulation. Mechanistically, bile acids such as TCA are known to enter cells via specific transporters, including the apical sodium-dependent bile acid transporter (ASBT) and sodium taurocholate cotransporting polypeptide (NTCP). Once internalized, they can activate nuclear receptors (e.g., FXR) and G-protein-coupled receptors (e.g., TGR5), thereby regulating downstream gene expression [55]. This provides a plausible explanation for the regulatory role of TCA observed in our study. As a natural bile acid, TCA exhibits significant clinical potential for treating UC [56]. Preclinical studies have demonstrated its anti-inflammatory effects through modulation of gut barrier integrity and immune responses [57]. Due to its natural origin and favorable safety profile, TCA represents a promising therapeutic candidate for UC with high translational potential. TGM2 plays a key role in immune regulation, extracellular matrix remodeling, and other pathophysiological processes, with its expression markedly altered in UC patients [58]. Given its critical involvement in inflammatory pathways, TGM2 shows promise as a potential biomarker for early UC diagnosis or disease activity monitoring. However, further clinical studies are required to validate its diagnostic sensitivity and specificity.

Another important point to address is the variation in predictive performance across different datasets. While our models achieved near-perfect results in the training set and in one independent validation cohort (GSE73661), the performance was lower in another external dataset (GSE87466), particularly in terms of specificity for some algorithms. This discrepancy is unlikely to result from simple model overfitting, as we employed repeated 10-fold cross-validation during training and used two independent validation datasets to evaluate model generalizability. Instead, the observed differences may reflect intrinsic heterogeneity among datasets, including variations in sample size, patient characteristics, and sequencing platforms, which can introduce biological and technical variability. Importantly, the consistently high AUC values across datasets suggest that the selected hub genes retain robust predictive power, even though classification performance varied between cohorts. This heterogeneity highlights the importance of validating predictive models in multiple independent cohorts and underscores the need for larger, multi-center studies to further strengthen the robustness and translational potential of the proposed model.

This study has several limitations that should be considered when interpreting the results. First, although we used in silico approaches such as network pharmacology and machine learning, these analyses primarily rely on existing public data and predictive models. These results still require wet lab validation. For example, the effects of TCA on ulcerative colitis and the role of TGM2 as a target need further experimental validation in animal models and clinical samples. Therefore, future studies should combine our computational findings with experimental data from animal models and human clinical samples to validate the therapeutic effects and underlying mechanisms of TCA. Additionally, our study lacks validation using human samples, and some of the data used were from animal models. Although these results provide preliminary evidence for the potential application of TCA in UC, clinical translation will require validation in human samples. Thus, future research should focus on the application of TCA in human ulcerative colitis patients, particularly validating it across different genders, age groups, and UC subtypes.

## 5. Conclusions

**Figure 9:**
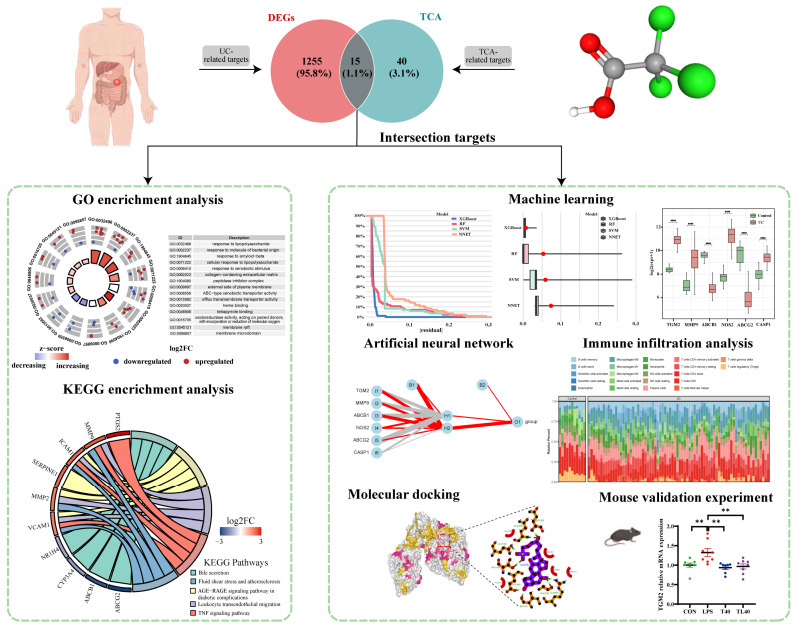
Network pharmacology regulatory mechanisms of taurocholate in anti-colitis. ** *p* < 0.01, **** *p* < 0.0001.

In a word, this study successfully predicted the DEGs of taurocholate target genes, ulcerative colitis, and normal samples by using network pharmacology, machine learning, immune infiltration, and molecular docking methods. Four machine learning models and neural network models were constructed for 15 intersecting genes, and six key characteristic genes were locked, namely *TGM2*, *MMP9*, *ABCB1*, *NOS2*, *ABCG2,* and *CASP1*. Following immunoregulatory interactions between a lyric and a non-lyric cell biological process, the infiltration of CD8 T cells and T cell regulation (tregs) was found to be high and significantly different, and negatively correlated with the *TGM2* gene. Molecular docking further confirmed that TGM2 proteins have the strongest binding affinity with taurocholate. The findings were further confirmed by experiments on mice. The analysis results of this paper point out that taurocholate has a therapeutic effect on ulcerative colitis, which provides theoretical guidance for further experimental and clinical research (Figure 9). However, the key proteins and predicted signal pathways need to be further verified by experiments.

## Data Availability

The data will be available upon request.

## Supplementary Materials

The following supporting information can be downloaded at: https://www.mdpi.com/article/10.3390/genes16091024/s1: Table S1: Information of the TCA’s targets, differentially expressed genes, and the intersecting targets. Table S2: The primer sequences of the six target genes. Table S3 Performance metrics of machine learning models on training and validation datasets. Table S4 Molecular docking cavities of TCA with each target protein.
